# Estimating the Effective Permittivity for Reconstructing Accurate Microwave-Radar Images

**DOI:** 10.1371/journal.pone.0160849

**Published:** 2016-09-09

**Authors:** Benjamin R. Lavoie, Michal Okoniewski, Elise C. Fear

**Affiliations:** Department of Electrical and Computer Engineering, Schulich School of Engineering, University of Calgary, Calgary, Alberta, T2N 1N4, Canada; Universidad Miguel Hernandez de Elche, SPAIN

## Abstract

We present preliminary results from a method for estimating the optimal effective permittivity for reconstructing microwave-radar images. Using knowledge of how microwave-radar images are formed, we identify characteristics that are typical of good images, and define a fitness function to measure the relative image quality. We build a polynomial interpolant of the fitness function in order to identify the most likely permittivity values of the tissue. To make the estimation process more efficient, the polynomial interpolant is constructed using a locally and dimensionally adaptive sampling method that is a novel combination of stochastic collocation and polynomial chaos. Examples, using a series of simulated, experimental and patient data collected using the Tissue Sensing Adaptive Radar system, which is under development at the University of Calgary, are presented. These examples show how, using our method, accurate images can be reconstructed starting with only a broad estimate of the permittivity range.

## Introduction

Near-field ultra-wide-band (UWB) microwave imaging techniques are under development as complementary methods to existing medical imaging techniques [1–10]. UWB radar can be used to map interior structures by exploiting differences in the electromagnetic properties of different tissues [11]. The resulting images show the locations of strong scatterers, which correspond to boundaries between tissues having a high electromagnetic contrast (e.g adipose and malignant tissue).

Microwave radar systems have attracted interest because of the potential for discriminating malignant tissue from surrounding material using a process that relies on different tissue properties than the existing techniques [12]. This allows the possibility of using microwave radar images to locate structures that could be missed by other imaging techniques. Furthermore, microwaves are non-ionizing, in contrast to X-rays used for mammography or computed tomography (CT) scans, and there is no need for a contrast agent, which is often used in magnetic resonance imaging. This means that multiple scans can be performed within short time intervals (weeks or even days). Work has been done with patient volunteers that has led to preliminary but promising results [5, 7].

Microwave radar imaging requires that the target be illuminated and the resulting reflections recorded by one or more antennas. Monostatic radar systems use a single antenna that acts as both a source and its own receiver. Multistatic radar systems use an array of antennas, and each acts, in turn, as a source with the rest acting as receivers. Both monostatic and multistatic systems are currently being developed for medical microwave imaging [5–7].

Constructing images from data obtained using either system requires an estimate of the electrical properties of the tissue (i.e. permittivity and conductivity). Multistatic radar has the ability to estimate the average values of the electrical properties between antenna pairs using time-of-flight measurements [13], and some work has been done to create a velocity map from this data [14]. Monostatic radar, however, cannot perform such measurements so the tissue properties must be obtained in another way. One possible method is to perform a separate measurement, using a pair of antenna arrays, to estimate the average properties [15]. In either case, the image obtained must be assumed to be the correct image, as there is no way to determine if the permittivity estimate is incorrect. Incorrect permittivity estimation can have a detrimental effect on the reconstructed images and, therefore, can lead to poor image accuracy and consistency, or even false detections.

We propose a method for estimating the effective tissue properties for monostatic radar imaging that leverages knowledge of how the microwave radar images are reconstructed. From the image reconstruction technique, we determine how the images depend on the estimated tissue properties. From this, selected image parameters are identified as important, and are used to construct a fitness function that reflects the quality of the image. A “good” image (one with high fitness) would correspond to the correct effective permittivity values with a high probability. In this way, we are building a kind of confidence map for the possible permittivity values. A similar technique was recently published with preliminary results for detecting strokes [9]. Our method differs from this method in two key aspects, which will be discussed.

We calculate the fitness function for a large number of images, which are generated using test values for the tissue permittivities. The fitness function is then reconstructed using a polynomial interpolant that is fitted between nodes where the fitness function was calculated explicitly. As the number of images to be reconstructed is large, it is important to choose the test values efficiently. Therefore, we employ a unique combination of adaptive stochastic collocation and polynomial chaos methods to choose the test values. From the reconstructed fitness function, we can obtain the permittivity values that provide the best image, which is also an estimate of the actual effective tissue permittivities.

## Approach

### Determining Image Quality

In microwave radar systems, reflection data is recorded after exciting the antenna and then recording the subsequent reflections that are scattered back to the antenna aperture. Reflections from the scanning apparatus itself are eliminated by subtracting the results of a calibration scan (one conducted with no patient present) done with an identical set of antenna positions. Reflections from the patient’s skin are eliminated using a signal filtering technique [2, 16], which also tends to remove reflections from glandular tissue.

The filtering is done for each signal (target) by, first, selecting a number of signals from nearby antenna positions, which are chosen based on proximity and similarity to the target signal. The start and end times of the primary skin reflection are then identified in the target signal and used, along with the adjacent signals, to construct a filter. The skin response is then removed from the target signal by applying the filter. This process is repeated for each recorded signal.

The tissue sensing adaptive radar (TSAR) system [1, 2, 4, 5], which is being developed at the University of Calgary, is used to collect data and reconstruct the microwave radar images in this paper. The imaging method uses the time taken for a pulse to reach a scatterer, partially reflect, and return to the antenna, to estimate the distance from the antenna to the scatterer [1]. This requires knowledge of the tissue permittivity.

Two issues arise when converting time data to spatial data. First, the time data does not contain information about the direction, only round-trip time of the signal. This means that the location of scatterers is not resolvable from a single signal. Second, the signal speed is required for proper image formation.

**Fig 1:**
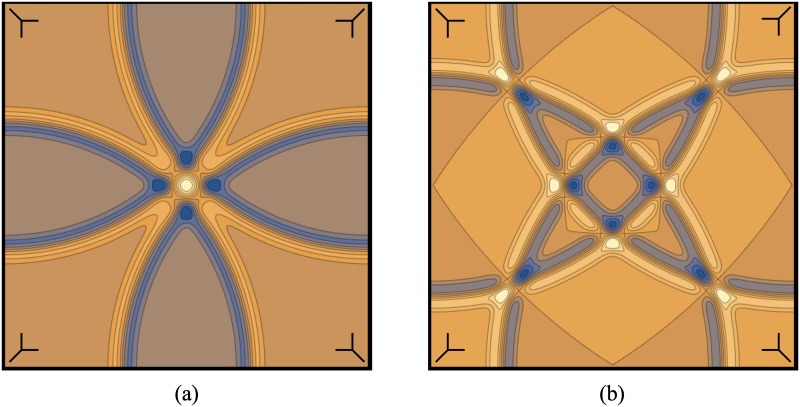
Exapmle of properly and improperly aligned signals. An example of simplified 2D signals (a) combined with a good signal speed estimate and properly aligned, and (b) combined with a poor signal speed estimate and misaligned. The misaligned signals clearly generate a number of artifacts. As more signals are added, the compounded effect of constructive interference will lead to significantly more contrast between the two cases. The antenna positions are indicated in the corners of of each plot.

The first problem is dealt with by using multiple antenna positions with overlapping acceptance angles. When the spatial signals are then combined to form an image, corresponding peaks in the signal will interfere constructively where scatterers are located, and destructively in other areas. However, in order for this to work properly, the signal speed must be known. If the estimated signal speed is incorrect, then the signals will not combine in the correct way. Fig 1 shows how the signals combine with both good and poor signal speed estimates.

Generally, both the skin and interior tissue are treated as homogeneous and lossless, so real-valued permittivities are used [5, 7]. Even so, determining the appropriate signal speed for image reconstruction is not straight forward, as the tissues cannot be accessed directly to measure the permittivity. Average values can be obtained, but these are not always representative of the actual signal paths. We provide a way to estimate the effective tissue permittivity by calculating the fitness of individual images formed with different permittivity estimates.

We use the term effective permittivity, because we are not estimating the actual average permittivity. Each signal used to illuminate the breast from a different position experiences a unique average permittivity that is determined by the type and amount of tissues that it passes through. What we are estimating is the permittivity value that best represents a weighted average of these individual permittivities, which we call the effective permittivity. The weighting is not done explicitly, but is implicit in the image reconstruction, as signals that are attenuated less will contribute more to the final image. Therefore, we expect the signal paths that are shorter or travel through tissue with lower conductivity to have a higher weight in the effective permittivity.

**Fig 2:**
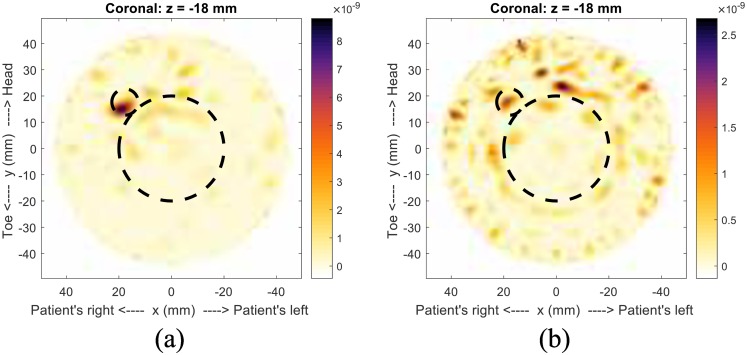
Examples of good and poor images. A comparison of images formed with simulated data. (a) is an image formed with a good permittivity estimate, clearly showing the presence of a scatterer on the left of centre. The dimmer second response is an artifact due to simulated glandular tissue in the centre. (b) is an image formed with a poor permittivity choice. A cluster of artifacts is present in the upper left, with almost no response where the scatterer is actually located. The small and large dotted circles represent the actual locations of the tumor and glandular tissue, respectively.

When an image is constructed with an incorrect permittivity estimation, the interference pattern from combining the signals tends to develop several general characteristics. As the permittivity estimate is moved away from the actual value, the signal peaks will not be properly centred on the scatterers. As a result, the response in the image will tend to be weaker and broader than the response for the correct permittivity values. If the estimated permittivity is further from the correct values, the constructive interference of signals in incorrect locations can create a number of artifact peaks. Fig 2 shows a comparison of images formed with good and bad permittivity estimates.

A good image will generally have fewer peaks a small area of constructive interference and a maximum peak that is higher. The figures in S1 Fig show how the permittivity estimation affects image quality. The simplest function that captures the requirements for a good image is

$$f_{1}=\frac{S_{\text{max}}}{NA},$$ (1)

with *S*_max_, the maximum of the absolute value of the image, *A*, the total area in the image with absolute pixel values greater than *S*_max_/2, and *N* the number of distinct areas that contribute to *A*. The absolute value of the response is used, because in some images constructive interference of the negative scattering response is more representative of the actual structure.

For some values of the permittivity, small and very bright artifacts appear at the edges of the image. Therefore, we include a function that penalizes images with *S*_max_ too close to the edge, so the fitness function becomes

$$f=f_{1}P,$$ (2)

with

$$P=\min\left({\left(\frac{d}{3\text{mm}}\right)}^{3},1\right),$$ (3)

where *d* is the distance from the image boundary to the pixel corresponding to *S*_max_. We use a penalty distance of 3mm to account for an average skin thickness of 2mm plus a 1mm buffer.

The fitness function was developed for 2-D images, which are generated using 3-D data and then sliced at the known location of the tumor response. This was done in order to use simpler images to test our metric. The implementation of our metric can be extended for use with 3-D images by replacing the response area, *A*, with the response volume. Most of the results that will be presented use the fitness function for 2D images, but we do present two cases showing preliminary 3D results.

This fitness function is biased toward images that indicate some “response”, whatever the source. This assumption, however, is not unreasonable, as healthy patients will have some amount of glandular tissue to scatter the signals. Thus, a response will still be present, but will result from signals scattering off glandular tissue, rather than a tumor. Furthermore, we would expect to see a response even if the images were reconstructed with the correct permittivity, as the individual reflections would add coherently.

Natural variations in breast tissues (e.g. the amount and distribution of glandular tissues and variations in breast size), along with unpredictable tumor locations, prevent the use of a reference image with which to assess the quality of an image. As a result, there is no “optimal” function with which to compare the quality of images, so one must be chosen. To avoid arbitrarily choosing a fitness function, we developed the above framework to identify what properties would likely be important.

We tested a number of variations that were inspired by our experience with the imaging method and system, and an analysis of the images with changing permittivity. Our final choice of fitness function performed the best overall for a variety of test cases. In addition to variants on functions similar to Eq (2), we tested fitness functions based on Laplace filters and Fourier transforms, to see if some underlying features could be linked to image quality. The results from functions based on these methods was inconsistent, unfortunately.

The objective now is to identify the permittivity values that maximize Eq (2). However, in order to calculate the fitness, an image needs to be generated for each test permittivity. While the use of fast reconstruction techniques has reduced the image reconstruction time to as little as 12s for 3D images [17], a large number of images is required to construct the fitness function. We now describe our method for efficiently determining the permittivity estimate corresponding to the maximum fitness.

### Permittivity Estimation Method

Efficiently determining the correct permittivity values that correspond to the maximum value of the fitness function requires an appropriate strategy. First, the convergence of the strategy needs to be weakly dependent on an initial guess, as the range of possible permittivity values is large [11] and no other information about the permittivities is known beforehand. Second, the strategy needs to be able to efficiently cover the entire parameter space and return multiple possible solutions, as there could potentially be false positives due to artifacts. Finally, to improve efficiency, the method should be able to adaptively sample the parameter space, as we expect to see relatively small areas of interest (high fitness).

Two similar strategies are well suited to address the requirements of our estimation method: stochastic collocation (SC) [18, 19] and polynomial chaos (PC) [20, 21]. Both of these approaches are methods for building a polynomial approximation of a function of multiple parameters. As SC tends to converge with fewer samples than PC [22], we use it as our primary approximation method. One drawback to adaptive SC, however, is its inability to determine the sensitivity of the interpolant to the parameters being tested. PC provides this information in the expansion coefficients when particular subsets of them are summed [23–25], so we take advantage of this fact by building a PC approximation of the constructed polynomial interpolant. We can then compute the sensitivity indices for each parameter and decide which parameters should be included when choosing new sample nodes at each stage.

The main polynomial approximation uses the hierarchical SC method, which fits Lagrange polynomials between data nodes [18, 19]. The interpolant is refined using the hierarchical surpluses, which are the differences between the previous interpolant and the actual function at a new set of nodes. The hierarchical surpluses can also be used as an estimate of the accuracy of the interpolant [19].

**Fig 3:**
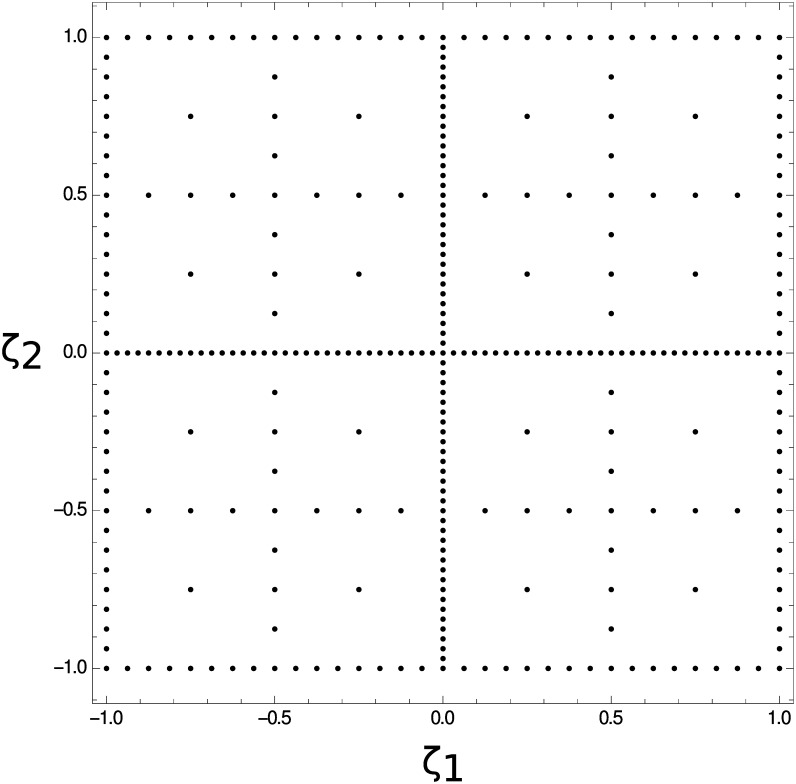
A level 6 Smolyak sparse grid with 321 nodes.

The required data are collected at nodes on the so-called Smolyak sparse grid, as this sampling method provides reduced sample density (total number of constructed images) with minimal loss of accuracy [26]. Fig 3 shows a level 6 sparse grid, where the *ζ*_1_ and *ζ*_2_ are for the purpose of generating the grid, and are rescaled to fit the appropriate ranges of *ϵ*_*i*_ and *ϵ*_*s*_, respectively, before forming images and calculating the fitness. To further avoid unnecessarily long simulation times, our algorithm terminates after the interpolant has been built on the level 10 sparse grid, whether the interpolant is within error tolerance or not. This choice provides a reasonable compromise between the number of nodes and coverage of the parameter space, and does not negatively affect the final result, as the major features of the fitness function have been identified by this point.

The full level 10 sparse grid has 7169 nodes, but by adaptively choosing them we are able to construct our interpolants using between 1000–3000 images per model, while still maintaining high accuracy. We wish to note that these images are not the result of separate optimization procedures, but are simply constructed with different permittivity choices. The optimization procedure uses these images to estimate the best permittivity choice.

The adaptive SC method we use is similar to that devised by Ma and Zabaras [19]. This method, which is based on hierarchical SC, builds up the interpolation in stages, and adds more nodes in each stage where necessary until either an accuracy goal is met or a predetermined highest stage is reached. It should be noted that the number of nodes required does not, in general, increase with the dimensions of the image (e.g. from 2D to 3D), but rather with the number of parameters being optimized.

**Fig 4:**
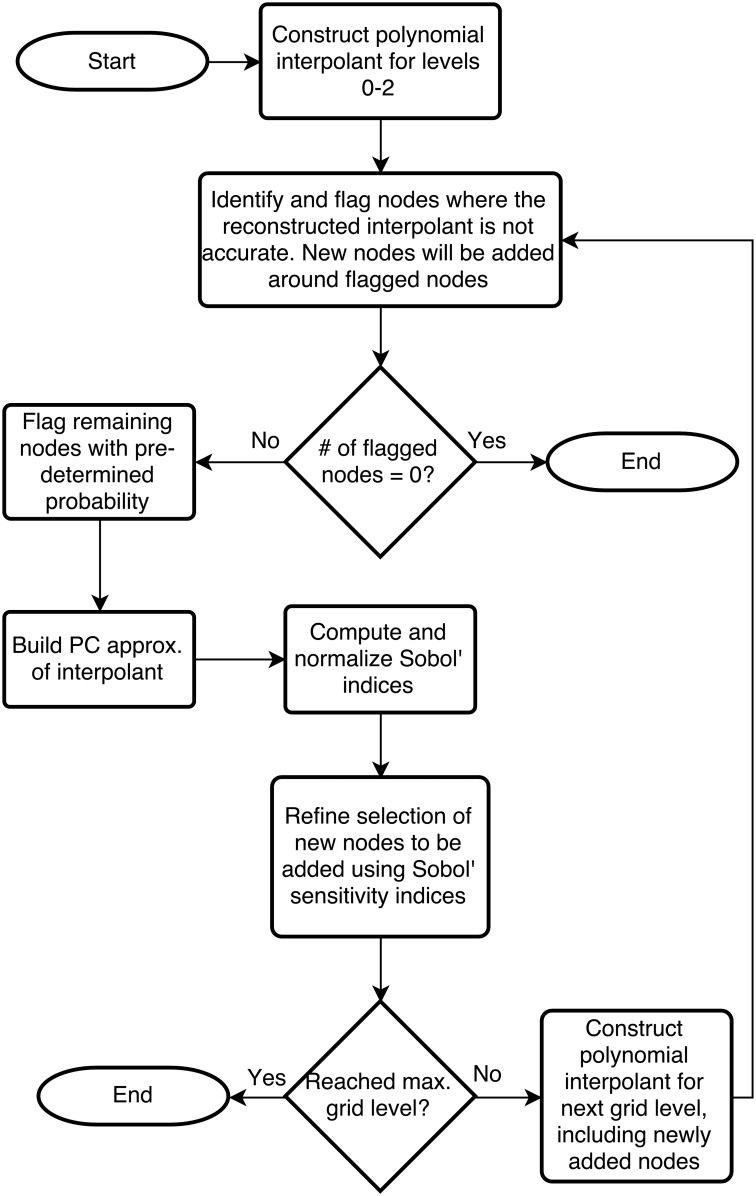
A flow chart for the optimization algorithm. A flow chart depicting the program for adaptively generating nodes on which the image fitness is calculated.

Our method works by, first, constructing the polynomial interpolant using non-adaptive SC for the first three stages (levels 0–2). We begin with a non-adaptive method to allow enough initial nodes to be sampled for the adaptive method to be reliable. After the nodes in level 2 have been computed, we can begin adaptively selecting new nodes. Fig 4 shows a flowchart that outlines the process for adaptively choosing nodes.

The first step to generating a new set of nodes is to compare the hierarchical surpluses of the nodes in the current level to a predetermined error tolerance (we chose 1% of the difference between the maximum and minimum values currently calculated from the actual function). If the hierarchical surplus is larger than the error tolerance, the node is flagged, and will be included in the next step. As the error tolerance depends on the known range of the function, it could increase as more function values are calculated.

If a node is not flagged, the change in the interpolant at that node over the previous two stages is computed, and if that change is large enough (we use 10 times the error tolerance mentioned previously) the node is flagged. This step is to catch any nodes where the interpolant is close at that node, but not accurate around the node (e.g. the interpolant crosses through the correct value at the node, but deviates elsewhere). At this stage, if no nodes have been flagged, the interpolant is deemed to be within the error tolerance, so the required information to reconstruct the interpolant is saved and the program is terminated. However, if at least one node is flagged, the program continues, and any nodes in the current grid level that are not yet flagged have a 20% probability of being included. This is to sparsely populate areas that would otherwise be left empty in order to prevent missed details.

To further refine our set of new nodes, we build a PC approximation of the current interpolant. From the PC approximation we compute the Sobol′ sensitivity indices to determine the sensitivity of the fitness function to each individual parameter [23–25]. Although we only use two parameters in this case, the method can be extended to a larger number of parameters.

We can now determine which nodes to include in the next grid level. To start we normalize the Sobol′ indices to 1 by dividing each one by the largest. Then, to determine if the neighbouring nodes of a flagged node should be added in a particular direction (corresponding to a parameter), the hierarchical surplus (or the change in the interpolant) is multiplied by the respective Sobol′ index. If the resulting value is larger than our error tolerance, the two neighbour nodes in that direction are added. In this way, local adaptivity is achieved by only including nodes that have a large enough error, and the dimensional adaptivity is achieved by using the Sobol′ indices to further refine the set of nodes to be added.

The new interpolant is now constructed, and the process is repeated until the interpolant is within the chosen error tolerance, or the maximum grid level is reached. We then plot the interpolant to identify areas of high fitness and determine the most likely permittivity values. These values are then used to reconstruct the microwave-radar image and identify the locations of strong scatterers.

## Results

In this section we present results obtained using our permittivity estimation method. Plots of Eq (2) as a function of the effective skin and interior tissue permittivities are presented, and images formed using the most likely permittivity values are compared with the known properties of the simulation/model, where available. To test the fitness function for 2D images, the first model we use is a simplified model with both simulated and experimentally collected data. The second model is a more realistic model, which is used to generate simulated data. Finally, patient data is used to test the fitness function under actual scan conditions. Additionally, we provide preliminary tests of a fitness function for 3D images using the simplified model and a another set of patient data. All of the fitness functions are normalized to unity, but use the same color scheme as the images for clarity.

The various models we use are chosen to highlight the capability of the estimation method to produce accurate and consistent images under various circumstances. Image quality is affected by a number of different factors [17, 27–31], but the dominant effect of permittivity choice is the image accuracy.

### Model #1—Simulations

Model #1 is a simplified cylindrical breast model that has a 2mm thick outer layer to represent the skin [32]. This skin layer is a tapered cylinder at 80^°^ to normal with a maximum diameter of 10cm that is joined to a hemisphere of matching diameter. Inclusions can be inserted to approximate glandular and malignant tissues. The skin layer and inclusions are constructed from different combinations of graphite, carbon black and urethane rubber to approximate the electromagnetic properties of the tissues they are representing. To simulate fatty tissue, the model was filled with canola oil, which has very low conductivity and dispersion at microwave frequencies, and has a relative permittivity of approximately 2.5. This model was used to generate both simulated and experimentally collected data.

The first example, Model #1-a, is a simulation of Model #1 with a single inclusion. The relative permittivities of the inner region, *ϵ*_*i*_, and skin layer, *ϵ*_*s*_, are 2.5 and 25, respectively. The inclusion, which is used to represent a tumor, is 10mm in diameter and located at *x* = 25mm, *y* = 0mm and *z* = −18mm.

**Fig 5:**
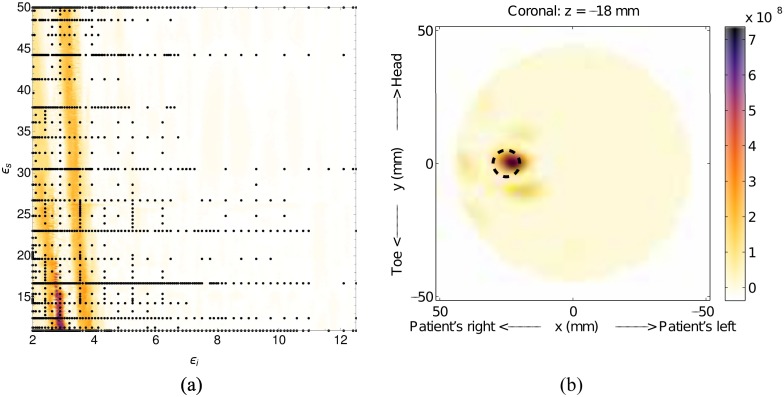
Results for Model #1-a. The fitness, (a), as a function of effective interior tissue permittivity, *ϵ*_*i*_, and effective skin permittivity, *ϵ*_*s*_, and (b) is the image formed using the most likely permittivity values, *ϵ*_*i*_ = 2.9 and *ϵ*_*s*_ = 12.2. The dashed circle in (b) represents the actual location of the scatterer.

The fitness function and the resulting image, which is a cross-section perpendicular to the cylinder axis and passing though the inclusion, for this simulation are shown in Fig 5. The region of high-likelihood in the fitness function is very localized and distinct, and has a maximum value at *ϵ*_*i*_ = 2.9 and *ϵ*_*s*_ = 12.2. The estimated effective interior permittivity of 2.9 is close to the actual value of 2.5. The estimate for the skin of 12.2 is quite low, but as the skin layer is very thin, its overall effect on the image is less significant. This, as will become apparent with more examples, makes estimating the skin permittivity difficult.

The image formed with the estimated parameters is very clean (free of artifacts) and the response is in the correct location (the dashed circle in Fig 5b), indicating that our method is good at accurately reproducing images of single scatterers. Constructing the fitness function took 1363 nodes, and the construction was terminated after the tenth grid level. It can be seen in Fig 5a that considerable computational savings, in terms of nodes sampled, were made in the upper right quadrant, where the fitness function is very flat.

**Fig 6:**
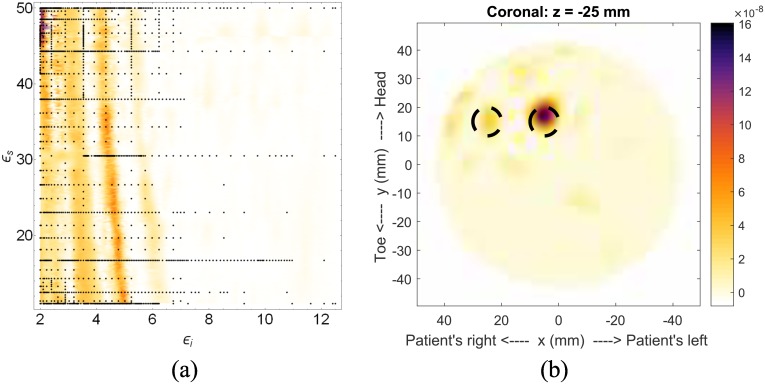
Results for Model #1-b. The fitness, (a), as a function of effective interior tissue permittivity, *ϵ*_*i*_, and effective skin permittivity, *ϵ*_*s*_, and (a) is the image formed using the most likely permittivity values, *ϵ*_*i*_ = 2.1 and *ϵ*_*s*_ = 46.3.

A second scatterer was added to Model #1 to form Model #1-b. The two scatterers, both 10mm in diameter, are located at *x* = 25mm, *y* = 15mm and *z* = −25mm, and *x* = 5mm, *y* = 15mm and *z* = −25mm. This case shows how the permittivity optimization deals with two scatterers. The fitness and highest-likelihood image are shown in Fig 6. It is clear from this case that multiple scatterers can cause issues, specifically because the fitness prefers images with fewer responses. However, this is less of an issue in actual scans, where noise creates artifacts that are more numerous and prominent, especially with incorrect permittivity choices.

**Fig 7:**
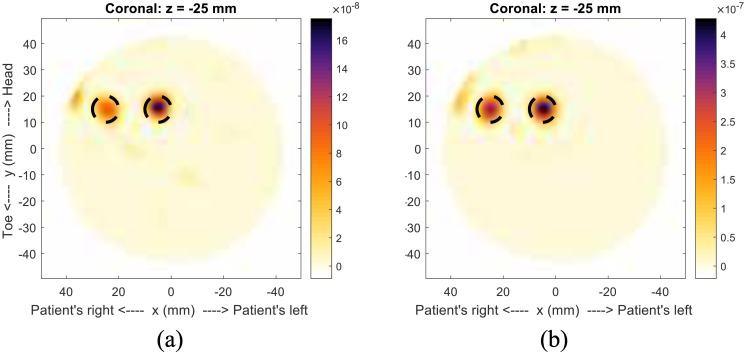
Model #1-b showing response degradation. Images of Model #1-b showing (a) how the response for the scatterer at *x* = 20mm has been degraded by the skin-suppression algorithm, when compared to (b) the image formed by removing the skin response using an identical scan, but without the scatterers.

Despite the potential issue with two scatterers, the most-likely permittivity values obtained from the fitness function are *ϵ*_*i*_ = 2.1 and *ϵ*_*s*_ = 46.3, which are similar to the actual values of *ϵ*_*i*_ = 2.5 and *ϵ*_*s*_ = 25. As a comparison, and to show how the skin-suppression algorithm can degrade responses, two images are shown in Fig 7. Both are formed with the actual permittivities, one with the skin-suppression algorithm and the other with subtraction of the tumor-free case. It is clear that the response from the outermost scatterer has been degraded with the skin-suppression algorithm.

**Fig 8:**
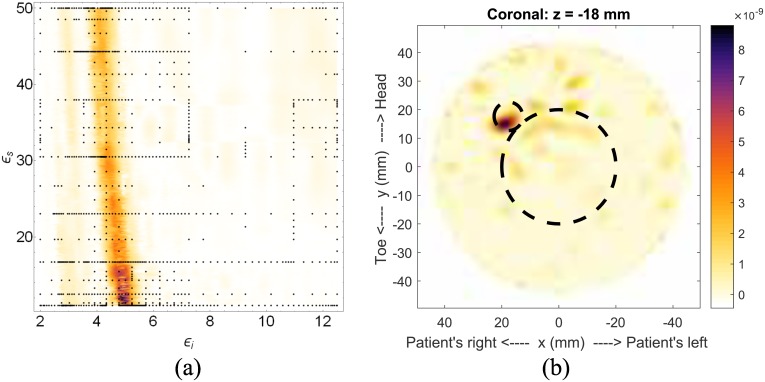
Results for Model #1-c. The fitness, (a), as a function of effective interior tissue permittivity, *ϵ*_*i*_, and effective skin permittivity, *ϵ*_*s*_, and (b) is the image formed using the most likely permittivity values, *ϵ*_*i*_ = 4.9 and *ϵ*_*s*_ = 11.8.

We now introduce a second, larger scatterer into the model to represent the presence of glandular tissue and shift the tumor to *x* = 17.68mm, *y* = 17.68mm and *z* = −18mm (Model #1-c). The simulated glandular tissue is a cylinder with 40mm diameter, located directly in the center of the simulation area. As with Model #1-a, the geometry is simple in order to assess the behaviour of the fitness function. The fitness function, shown in Fig 8a, has a slightly broader high-likelihood region than the previous simulation, but is still localized. The resulting image, Fig 8b, clearly shows the response from the tumor and the glandular tissue response has been removed by the skin-suppression algorithm [16].

The estimated effective permittivity values for this simulation were determined to be *ϵ*_*i*_ = 4.9 and *ϵ*_*s*_ = 11.8. The skin permittivity, again, is quite low, but consistent with the previous simulation. The estimated value of *ϵ*_*i*_ is greater than that for Model #1-a, which is expected, but it is difficult to assess the accuracy of this value. For reasons that will be discussed later, a volumetric average of the tissue permittivities would not be consistent with monostatic radar imaging. Constructing the fitness function for this example took 882 nodes, and the construction was terminated after the tenth grid level. As with the last case, very few nodes were spent generating images where the fitness function is flat.

**Fig 9:**
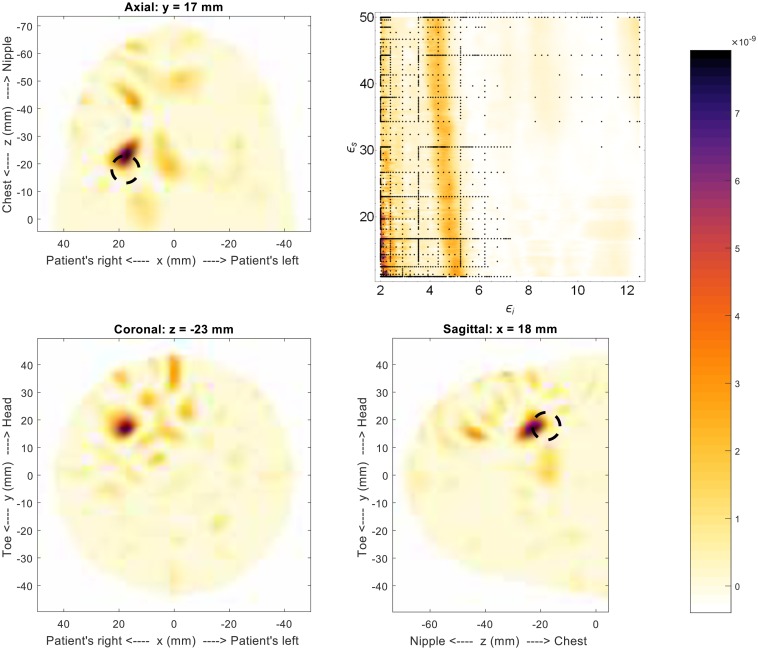
Results for 3D image of Model #1-c. The fitness, upper right, as a function of effective interior tissue permittivity, *ϵ*_*i*_, and effective skin permittivity, *ϵ*_*s*_, and 3D image generated using the permittivity values *ϵ*_*i*_ = 4.8 and *ϵ*_*s*_ = 23. This result is consistent with the 2D result and sketch, both shown in Fig 8. The dashed circle represents the location of the simulated tumor.

We now present preliminary results using a 3D version of the fitness function. This example uses the full 3D scan of Model #1-c, and the result is shown in Fig 9. The most-likely permittivity identified by the fitness function is very low (approx 2.1). This permittivity estimate is due to an artifact that is a direct result of the high level of symmetry in this model (the reflections from the glandular tissue were shifted to the centre, creating an artificially strong response). Looking at the secondary region of high-likelihood, a permittivity estimate of *ϵ*_*i*_ = 4.8 and *ϵ*_*s*_ = 23 is obtained and used to generate the image in Fig 9. The 3D result is consistent with the 2D result, showing a single scatterer very near the location of the simulated tumor. There is a slight discrepancy in the *z* position of the tumor, with this example placing it 6mm further from the chest wall than its actual location. But, overall, this result shows that the 3D fitness function can produce useful images.

### Model #1—Experiments

**Fig 10:**
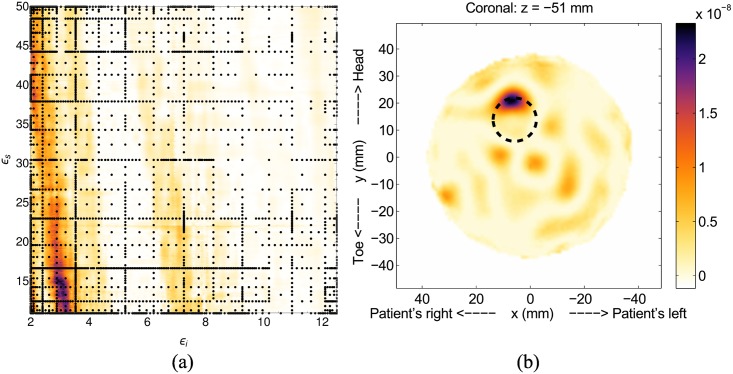
Results for Model #1-d. The fitness, (a), as a function of effective interior tissue permittivity, *ϵ*_*i*_, and effective skin permittivity, *ϵ*_*s*_, and (a) is the image formed using the most likely permittivity values, *ϵ*_*i*_ = 2.9 and *ϵ*_*s*_ = 15.1.

We now present results using experimentally collected data to test our method under more realistic conditions. The first experimental result, Model #1-d, is for a single 16mm diameter scatterer placed at *x* = 5.74mm, *y* = 13.9mm and *z* = −51mm. The fitness function, Fig 10a, has a very distinct and localized region of high-likelihood, with the maximum likelihood at *ϵ*_*i*_ = 2.9 and *ϵ*_*s*_ = 15.1. As with the simulations, the effective interior permittivity is close to the nominal value of 2.5 for canola oil, and the skin value is underestimated.

The image corresponding to the maximum likelihood point, Fig 10b, shows the scatterer clearly visible and located very close to the actual location, which is indicated by the dashed circle. Artifacts are present in the image, likely due to noise, but they are low intensity (about 1/2 that of the scatterer), so are easily distinguished from the scatterer. Construction of the fitness function for this case used 2914 sample nodes and was terminated after the tenth grid level.

**Fig 11:**
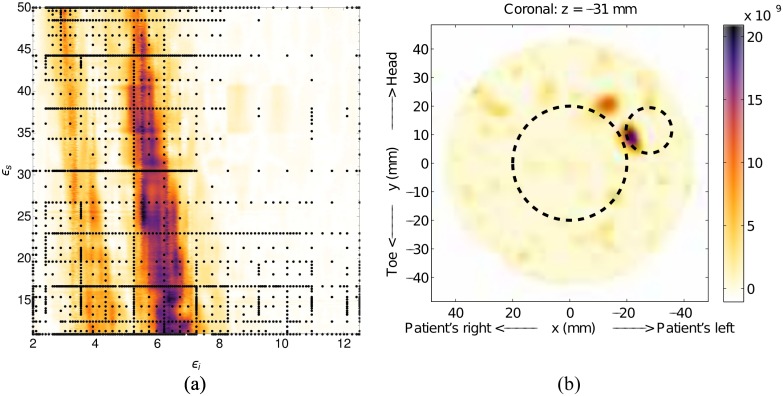
Results for Model #1-e. The fitness, (a), as a function of effective interior tissue permittivity, *ϵ*_*i*_, and effective skin permittivity, *ϵ*_*s*_, and (b) is the image formed using the most likely permittivity values, *ϵ*_*i*_ = 6.4 and *ϵ*_*s*_ = 15.

A 40mm diameter cylindrical scatterer, representing glandular tissue, was inserted into the centre of Model #1 to construct Model #1-e, along with a 16mm diameter scatterer, which represents a tumor, at *x* = −27.7mm, *y* = 11.5mm and *z* = −31mm. The region of high-likelihood, Fig 11a, for this case is not as well localized as the simulations, and has a maximum at *ϵ*_*i*_ = 6.4 and *ϵ*_*s*_ = 15. Despite the large high-likelihood region, the image generated using the maximum-likelihood point, shown in Fig 11b, clearly shows a single strong scatterer, along with some low-intensity artifacts.

Although the size of the response in Fig 11b is small, possibly due to the clutter reduction algorithm, the location corresponds well to that of the scatterer (white dashed circle). As with Model #1-c, the response from the gland is completely removed due to the skin-suppression algorithm, and the accuracy of the interior permittivity is difficult to assess. The fitness function for this example was constructed using 1972 sample nodes, and construction was terminated after the tenth grid level.

Model #1-e illustrates the effect of our skin-suppression algorithm on scatterers that are near the skin; the response is degraded, though still located at the correct location. Smaller scatterers are affected less, as there will be less similarity between signals from neighboring antennas. While this may seem like a limitation of the imaging system, in a clinical setting, tumors that are situated near the skin are generally palpable with a physical exam, and can affect the skin appearance (known as peau d’orange) [33]. Due to these factors, complementary imaging would not be needed for tumors near the skin.

The estimated effective interior permittivities are consistent between the simulations and experimentally acquired data. Although the estimates for the skin permittivity vary, it has a much smaller impact on the final image than the interior permittivity. Additionally, the fitness functions also agree well, with those for the experimental data having slightly reduced precision, which is likely due to the presence of noise. With the tests on simple geometries showing very promising results, we now increase the complexity of the model to assess the effect of irregular geometries.

### Model #2

Model #2 is an increasingly realistic breast model based on magnetic resonance imaging scans, and scanned with a patient-specific geometry [31]. All of the examples that use this model are simulations to allow more freedom in choosing irregular geometries, particularly for the glandular tissue. Additionally, for simulating the imaging process, the tissue permittivities in this model were chosen to be more representative of actual human tissue, including the effects of dispersion. The relative permittivity used for the fatty (or background) tissue is 4.64 at 4GHz (approximately the center frequency of the pulse). This basic model consists of an outer skin layer surrounding fatty tissue, and is used to construct the more detailed models that we use tested.

**Fig 12:**
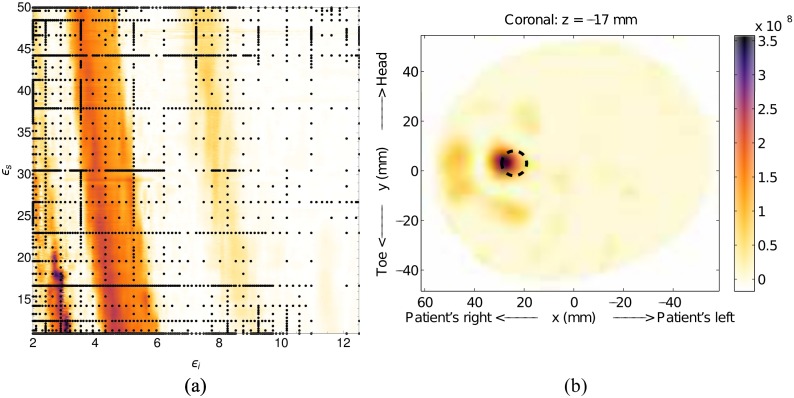
Results for Model #2-a. The fitness, (a), as a function of effective interior tissue permittivity, *ϵ*_*i*_, and effective skin permittivity, *ϵ*_*s*_, and (b) is the image formed using the most likely permittivity values, *ϵ*_*i*_ = 2.9 and *ϵ*_*s*_ = 17.8. The dashed circle represents the location of the tumor.

Model #2-a consists of a single 10mm diameter scatterer located at *x* = 24mm, *y* = 3mm and *z* = −17mm embedded in the basic model. The fitness function, Fig 12a, highlights the need for an estimation method that provides full coverage of the parameter space, rather than a single best guess; It is possible to have multiple regions of high likelihood that correspond to different permittivity estimates. In this case, the images constructed using the permittivities from both regions are very similar, but this may not be true for more complicated cases, such as actual patient scans. The fitness function for this model was constructed using 2381 nodes, and the program was terminated after the tenth grid level.

The image constructed from the maximum-likelihood point of *ϵ*_*i*_ = 2.9 and *ϵ*_*s*_ = 17.8 is shown in Fig 12b, and shows a clear response that is very near the correct location. The secondary high-likelihood region has a maximum of *ϵ*_*i*_ = 4.6 and *ϵ*_*s*_ = 13.5, which is much closer to the actual values. The image constructed with these values places the scatterer closer to the actual location, but the absolute response is weaker, which is why this image has a lower fitness.

**Fig 13:**
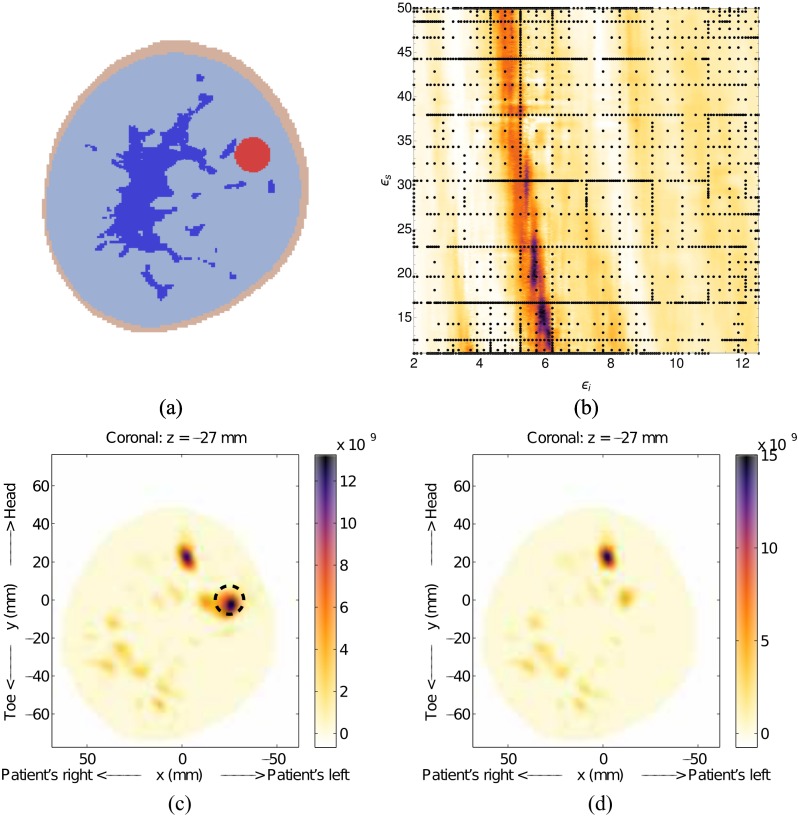
Results for Model #2-b. A sketch of the model (a), the fitness, (b), as a function of effective interior tissue permittivity, *ϵ*_*i*_, and effective skin permittivity, *ϵ*_*s*_, (c) is the image formed using the most likely permittivity values, *ϵ*_*i*_ = 6.1 and *ϵ*_*s*_ = 13.2, and (d) is an image generated using simulations done without the tumor. The tumor in (a) is represented by the small solid circle near the 3 o’clock position, whereas the gland is represented by the larger, irregularly shaped region at the center. The dashed circle in (c) indicates the tumor location.

Typically multiple, irregularly shaped scatterers are present in breast tissue, so we now introduce glandular tissue that is more representative of this. This example, Fig 13, is a comparison of two simulations with Model #2 geometry, having glandular tissue with an irregular geometry, leading to a heterogeneous interior. A direct comparison allows us to test the sensitivity of the imaging and estimation methods to differences in tissue properties.

The glandular tissue for the comparison is the same shape and in the same location, but has slightly different relative permittivities, 38.4 at 4GHz for Model #2-b and 34.7 at 4GHz for Model #2-c. A slice of the simulation space is shown in Fig 13a to show the tumor, having a 15mm diameter and located at *x* = −25mm, *y* = 0mm and *z* = −27mm, as well as the irregular glandular tissue and skin surface.

The high-likelihood region for Model #2-b, Fig 13b, is well localized, and a similar result is seen for Model #2-c. The most likely effective interior permittivity is estimated as 6.1 for Model #2-b and 5.9 for Model #2-c. The small difference is consistent with the fact that the permittivity of the glandular tissue in Model #2-b is slightly higher than that of Model #2-c. The image generated with the maximum-likelihood permittivity for Model #2-b, Fig 13c, shows the dominant responses in the correct location, indicated by the dashed circle. Construction of the fitness functions for Models #2-b and c took 2076 and 2029 nodes, respectively, and both were terminated after the tenth grid level.

The skin subtraction algorithm can remove large regular structures from the interior, so the response from the glandular tissue is largely removed from the image. However, there are two responses visible in the maximum-likelihood image, one is from the tumor and the second (located near 1 o’clock in the image) is due to the glandular tissue. The sketch in Fig 13a shows that a small portion of the glandular tissue extends up to where the second response is located. To further show this, an image generated using a simulation without the tumor and just the glandular tissue is shown in Fig 13d, which clearly shows the same glandular-tissue response as the maximum-likelihood images. A similar result was obtained for Model #2-c.

The previous examples have shown our method is capable of locating scatterers in the presence of heterogeneous interior tissue. However, these examples still do not adequately replicate the variations that naturally occur in breast tissue. We address this by testing our method on data collected during scans of patient volunteers.

### Patient Scans

The next two examples use patient scans to test the ability of our method to estimate tissue properties under actual scan conditions. The patient studies were approved by U of C Conjoint Health Research Ethics board, E-22121 and E-24098, and individual written patient consent was obtained. The prototype scan system [5] consists of a padded table, on which the patient lies prone with the breast to be scanned extending through a hole into the scanner. The sensor is a BAVA-D antenna [34], which operates from 2.4 GHz to 15 GHz. The antenna can be moved vertically and 360 degrees around the breast to acquire data from a number of perspectives. A scan of one breast typically lasts less than 30 minutes and with the patient lying prone, movement is not generally an issue.

**Fig 14:**
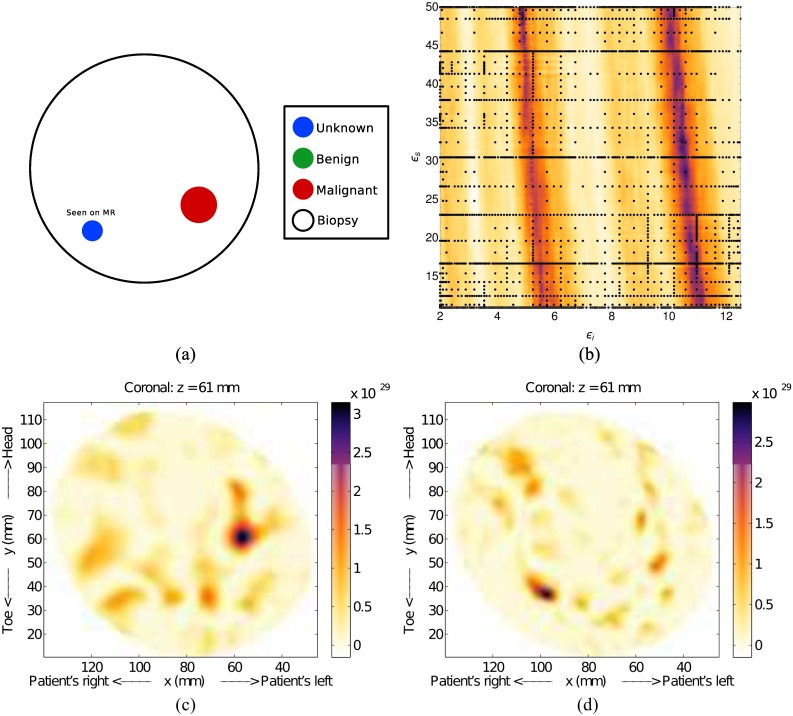
Results for Patient 4. The diagram representing the pathology reports (a), the fitness, (b), as a function of effective interior tissue permittivity, *ϵ*_*i*_, and effective skin permittivity, *ϵ*_*s*_, (c) is the image generated using the most likely permittivity values *ϵ*_*i*_ = 4.9 and *ϵ*_*s*_ = 49, and (d) is the image corresponding to the second region of high-likelihood (rightmost) and has *ϵ*_*i*_ = 10.5 and *ϵ*_*s*_ = 32.5.

The first example was included as Patient 4 in a previous study [5]. For that study, the pathology report was used to assess the accuracy of the images, and so a simple diagram was constructed to better visualize the information contained in the report. We will use this diagram, shown in Fig 14a, to assess our highest likelihood images. We show a 2D slice, selected to coincide with the response location in the previous study, for consistency with the previous results.

The fitness function for this example, shown in Fig 14b, has two clear and distinct regions that could correspond to the correct permittivity values. The image in Fig 14c was generated with the most likely permittivity values of *ϵ*_*i*_ = 4.9 and *ϵ*_*s*_ = 49, and the response at approximately the 3 o’clock position corresponds well with the malignant mass identified in the pathology diagram (Fig 14a). Construction of the fitness function took 2493 nodes and was terminated after the tenth grid level.

A second high-likelihood region is present in the fitness function, and the image that corresponds to the highest likelihood in this region is shown in Fig 14d. The malignant mass is not visible in this image, but there is a significant response at about the 7 o’clock position. The MR scans of this patient identified an unknown lesion in the same region, which is shown in the pathology diagram. In an image generated with permittivity values that are averages of the two most likely pairs, there are two responses visible that correspond to the two regions indicated on the pathology diagram. However, the responses are weaker and the image is noisier (more artifacts), so images in this region are poor according to our metric.

**Fig 15:**
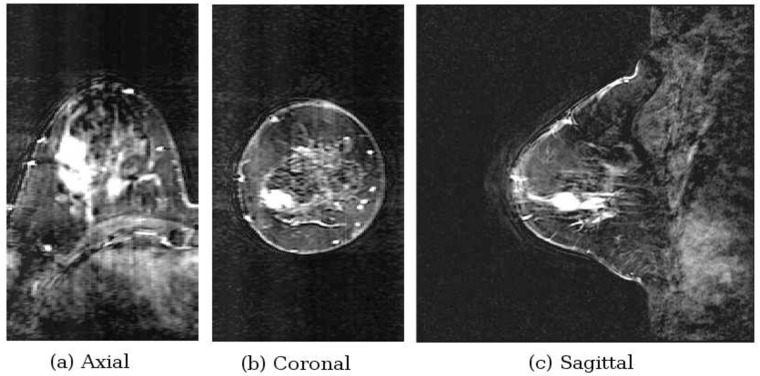
Contrast enhanced MR images for Patient 130920. This image shows the presence of a tumor (bright white region).

The consistency between the 2D and 3D results for Model #1-c lends confidence to the 3D fitness function, so we present a second 3D example, Patient 130920 from an ongoing patient study. This patient has a malignancy in her right breast. From the contrast enhanced MR images, Fig 15, the tumor is the bright spot located at approximately the 8 o’clock position in the coronal image.

**Fig 16:**
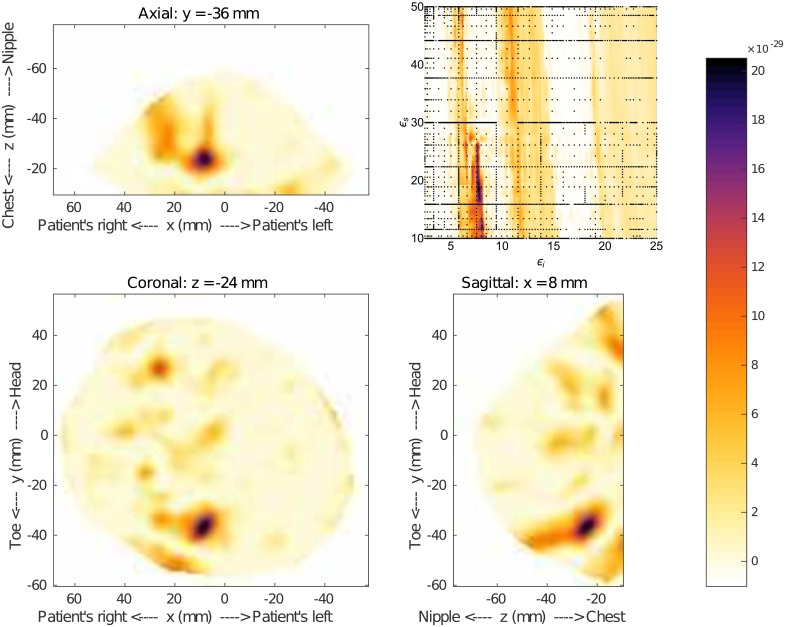
Results for Patient 130920. The fitness, upper right, as a function of effective interior tissue permittivity, *ϵ*_*i*_, and effective skin permittivity, *ϵ*_*s*_, and 3D image generated using the most likely permittivity values *ϵ*_*i*_ = 7.8 and *ϵ*_*s*_ = 18.5. This result shows a response in a similar location to that shown on with the MR images in Fig 15.

When compared to the image formed using the maximum-likelihood point, Fig 16, there is good agreement. The tumor location is not exact, though the difference in breast position during the different scans can account for some of the discrepancy. The estimated permittivity values are *ϵ*_*i*_ = 7.8 and *ϵ*_*s*_ = 18.5. The fitness function was constructed with 2798 nodes, and construction was terminated after the tenth grid level.

## Discussion

The large number of images that our method needs to generate is a necessary trade-off for having knowledge of the entire permittivity search space. The adaptive nature of our method is able to reduce the number of images, but sufficiently many are still needed to have confidence in the results. A smoother fitness function will require fewer adaptively chosen points than one that changes more rapidly with permittivity. As can be seen from S1 Fig, even relatively small permittivity changes can have significant effects on the image.

Reconstructing the images can be done quickly (less than 10 seconds per image). As the images are independent of each other, they could be constructed in parallel, thereby significantly decreasing the time required for the optimization process. Additionally, by better estimating the permittivity range, using the average permittivity as a guide, for instance, the number of required images could be reduced.

The high-likelihood regions in the fitness functions tend to be narrow. This implies that the average permittivity of the interior tissue has more influence on the image structure than that of the skin layer, which is unsurprising, as the skin layer is significantly thinner than the interior region. As a result, our method has difficulty estimating the permittivity of the skin, and tends to under estimate it. It may be possible to improve the skin permittivity estimation by fine tuning the fitness function, or simply obtain an independent estimate of the skin properties.

This study was done assuming the tissues are non-dispersive and lossless (i.e. constant permittivities). While this may seem like a very restrictive assumption, in practice we see little difference between images generated with this assumption and those using a Debye model. The benefits of assuming constant permittivities are that image formation is quicker and only two parameters need to be optimized (the skin and interior permittivities), rather than eight (four Debye parameters for each of the skin and interior tissue, or more if more than one pole is assumed). If, however, more information about the electrical properties of the tissue is desired, such as conductivity, our estimation method can certainly be used to obtain the Debye parameters. One drawback to assuming a constant permittivity, as mentioned earlier, is that we are approximating the permittivity as *ϵ*′ ≈ *n*′^2^, which is not strictly true for real dispersive materials.

Additionally, as we assume real permittivity values for the image reconstruction, the value that we estimate is actually the real part of the refractive index, *n*′. This is due to the fact that signal speed is determined by *n*′, which is defined by

$$n^{\prime }=\frac{1}{\sqrt{2}}\sqrt{\sqrt{\epsilon^{\prime 2}+\epsilon^{\prime \prime 2}}+\epsilon^{\prime }}$$ (4)

with *ϵ* = *ϵ*′ + *iϵ*′′ the complex permittivity. However, we expect the effective conductivity to be small compared to the effective permittivity, so we assume that $n^{\prime }\approx \sqrt{\epsilon^{\prime }}$. Thus, we expect our permittivity estimates to be slightly higher than the actual value.

An issue arose with the case of two scatterers (Model#1-b); the optimization method favored a permittivity that results in a single response, which is a consequence of attempting to reduce clutter. Additionally, one response has been degraded by the skin-suppression algorithm. As this microwave imaging is intended as a complementary modality (i.e. other images, such as mammograms, will be available), if more scatterers are suspected, but not imaged (possibly due to response degradation), the optimization can be run again with the already detected scatterers removed. This would give the weaker responses more weight in the fitness function, allowing them to be imaged.

Multiple high-likelihood regions appearing within a single fitness function generally results from one of three main causes. The first cause is simply artifacts due to incorrect permittivity estimation. Our fitness function seeks to eliminate this (our results show it generally does a good job), however, there could possibly be situations where an image showing an artifact, rather than a scatterer, would have a high fitness. This can be an issue in high-symmetry situations, such as the cylindrical phantoms (e.g. 3D image of Model #1-c).

The second cause of multiple high-likelihood regions is multiple strong scatterers (e.g. tumors), a situation that would not be unexpected as breast tissue is heterogeneous. In this case, signals with the strongest response from each scatterer experience a different effective permittivity than those for the other scatterers. As a result, when an image is reconstructed using the correct permittivity for one scatterer, the other scatterers become unfocused in the image. In such a case, multiple images can be produced, with each showing a different scatterer. Alternatively, reconstructing an image using a permittivity estimate that is somewhere in between may show some or all scatterers. Patient 4 is a good example of this, with the malignancy and unknown tissue appearing for different permittivity estimates.

The third reason is due to the pulse shape, which is the derivative of a Gaussian. As a result, it has a positive and negative peak that are nearly identical in magnitude. In some situations, such as with Model #2-a, different permittivity estimates cause the positive or negative peaks to interfere constructively, creating two very similar images and corresponding regions of high likelihood. This effect is actually present in Models #1-a, c, d and e, as well, only to a much lesser extent. The result is a characteristic “double-band” in the fitness function. This is in contrast to the distinct bands that appear for multiple scatterers, as for Patient 4.

Determining the correct effective permittivity for image reconstruction is a universal problem in microwave imaging. A method similar to that presented here is being developed to accurately reconstruct microwave images for stroke detection [9, 10]. This method differs from ours in three key aspects. First, their fitness function is designed simply to detect when there is significant contrast between one area of the image and the rest of the image, whereas ours tracks multiple image aspects. We tested a similar fitness function for reconstructing our images, but had inconsistent results.

The second difference between the two methods is how the maximum fitness is located. Our method uses a polynomial interpolant to estimate the fitness for every possible permittivity value. Their method uses particle swarm optimization to search for the maximum fitness. While using particle swarm optimization allows for optimizing more parameters simultaneously, it only recovers the global maximum, and has the potential to miss smaller features. We have found that ignoring all but the global maximum fitness can sometimes cause important features to be missed, for instance Model #2-a and Patient 4 in this study.

The third difference between the two methods is the number of estimated permittivity values. Whereas our method uses only two, their method assigns individual permittivity estimates to predefined signal-entry points, and assigns them to antenna-pixel pairs based on the shortest arrival time. Using multiple permittivity estimates in this way has the advantage of partially overcoming the homogeneity assumption. However, as the number of predefined entry points needs to be greater than the number of antennas [9], this approach cannot be applied directly with our method, as we would need more than 140 entry points. Furthermore, as we intend to extend this approach to 3-D images, the number of entry points would likely rise even further, so as to adequately cover the surface.

Future work for this method includes plans to address the assumption of a homogeneous interior region. Currently this assumption is made because we do not have knowledge of the types and distribution of the various tissues. While rough estimates could be made from MRI scans (if available), breast positioning is different for the two scan types, which would lead to discrepancies. Traditionally, a more accurate estimate of the tissue distribution would require inverse-problem techniques, significantly increasing the time for image formation. We are, however, currently working on a method to estimate the location of the glandular tissues from the radar data [35]. We are also considering other ways to address the issue of non-homogeneity, that allow us to keep the number of parameters to optimize small.

## Conclusion

Monostatic microwave-radar imaging systems require knowledge of the target’s permittivities in order to form an accurate image. This estimate can be directly obtained from time-of-flight measurements made with a separate measurement system. Such estimates, however, are not always representative of the effective permittivity experienced by the microwave signals. Rather than obtaining a direct estimate, we propose using image analysis to estimate the most likely effective permittivity.

The quality of each image generated is quantified using a fitness function that takes into account how microwave-radar images are constructed. By constructing a polynomial interpolant of the fitness as a function of the tissue permittivities, we can produce an image that reflects the actual locations of scatterers, along with an estimate of the effective tissue permittivities. The effective permittivity values are not representative of a volumetric average, but rather an average based on the paths taken by the various signals. Our method provides a means to identify the most likely effective permittivity values, along with the best image, thereby lending confidence to estimations of effective tissue permittivities, or eliminating the need for additional patient scans to estimate the permittivity altogether.

We tested our permittivity estimation method on various data sets imaged with the TSAR system, and the results, although preliminary, are very promising. Additional work is required to refine the fitness function, particularly for use with 3D images. More patient scans are needed to ensure our method is accurate for a wide range of tissues, and robust against noise and other sources of error, such as multiple strong scatterers. Scans of models with no malignant tissue, and healthy patients should be addressed, as well, to ensure accurate estimates and images can be obtained in these cases.

## Supporting Information

S1 Fig Effect of permittivity choice on image quality. These figures show how the quality of reconstructed images depends on the permittivity estimate used.
(PDF)

## References

[1] Fear EC, Li X, Hagness SC, Stuchly MA. Confocal microwave imaging for breast cancer detection: localization of tumors in three dimensions. IEEE Trans Biomed Eng. 2002 8;49(8):812–822. 10.1109/TBME.2002.800759 12148820

[2] Sill JM, Fear EC. Tissue sensing adaptive radar for breast cancer detection—experimental investigation of simple tumor models. IEEE Trans Microw Theory Techn. 2005 11;53(11):3312–3319. 10.1109/TMTT.2005.857330

[3] Nikolova NK. Microwave Imaging for Breast Cancer. IEEE Microw Mag. 2011 12;12(7):78–94. 10.1109/MMM.2011.942702

[4] Bourqui J, Sill JM, Fear EC. A Prototype System for Measuring Microwave Frequency Reflections from the Breast. Int J Biomed Imag. 2012;2012:851234 10.1155/2012/851234 PMC3348648 22611372

[5] Fear EC, Bourqui J, Curtis C, Mew D, Docktor B, Romano C. Microwave Breast Imaging With a Monostatic Radar-Based System: A Study of Application to Patients. IEEE Trans Microw Theory Techn. 2013;61(5):2119–2128. 10.1109/TMTT.2013.2255884

[6] Porter E, Kirshin E, Santorelli A, Coates M, Popovic M. Time-Domain Multistatic Radar System for Microwave Breast Screening. Antennas and Wireless Propagation Letters, IEEE. 2013;12:229–232. 10.1109/LAWP.2013.2247374

[7] Byrne D, Craddock IJ. Time-Domain Wideband Adaptive Beamforming for Radar Breast Imaging. IEEE Trans Antennas Propag. 2015 4;63(4):1725–1735. 10.1109/TAP.2015.2398125

[8] Santorelli A, Laforest O, Porter E, Popovic M. Image classification for a time-domain microwave radar system: Experiments with stable modular breast phantoms. In: Antennas and Propagation (EuCAP), 2015 9th European Conference on; 2015. p. 1–5.

[9] Guo L, Abbosh AM. Optimization-Based Confocal Microwave Imaging in Medical Applications. IEEE Trans Antennas Propag. 2015 8;63(8):3531–3539. 10.1109/TAP.2015.2434394

[10] Mobashsher AT, Bialkowski KS, Abbosh AM, Crozier S. Design and Experimental Evaluation of a Non-Invasive Microwave Head Imaging System for Intracranial Haemorrhage Detection. PLoS One. 2016;11(4):e0152351 10.1371/journal.pone.0152351 27073994 PMC4830520

[11] Lazebnik M, Popovic D, McCartney L, Watkins CB, Lindstrom MJ, Harter J, et al A large-scale study of the ultrawideband microwave dielectric properties of normal, benign and malignant breast tissues obtained from cancer surgeries. Phys Med Biol. 2007 10;52:6093–6115. 10.1088/0031-9155/52/20/002 17921574

[12] Curtis C, Frayne R, Fear EC. Using X-Ray Mammograms to Assist in Microwave Breast Image Interpretation. Int J Biomed Imag. 2012;2012:235380 10.1155/2012/235380 PMC3320031 22536208

[13] Sarafianou M, Craddock IJ, Henriksson T, Klemm M, Gibbins DR, Preece AW, et al. MUSIC processing for permittivity estimation in a Delay-and-Sum imaging system. In: Antennas and Propagation (EuCAP), 2013 7th European Conference on; 2013. p. 839–842.

[14] Deprez JF, Sarafianou M, Klemm M, Craddock IJ, Probert-Smith PJ. Defining Regions of Interest for Microwave Imaging Using Near-Field Reflection Data. Progress In Electromagnetics Research B. 2012 7;42:381–403.

[15] Bourqui J, Fear EC. Biological tissues assessment using transmitted microwave signals. In: Antennas and Propagation (EuCAP), 2014 8th European Conference on; 2014. p. 77–78.

[16] Maklad B, Curtis C, Fear EC, Messier GG. Neighborhood-based algorithm to facilitate the reduction of skin reflections in radar-based microwave imaging. PIER B. 2012;39:115–139. 10.2528/PIERB11122208

[17] Curtis CF, Fear EC. Near field radar imaging in the frequency domain with application to patient data. In: Radio Science Meeting (Joint with AP-S Symposium), 2015 USNC-URSI; 2015. p. 306–306.

[18] Agarwal N, Aluru NR. A domain adaptive stochastic collocation approach for analysis of MEMS under uncertainties. J Comput Phys. 2009;228(20):7662–7688. 10.1016/j.jcp.2009.07.014

[19] Ma X, Zabaras N. An adaptive hierarchical sparse grid collocation algorithm for the solution of stochastic differential equations. J Comput Phys. 2009;228:3084–3113. 10.1016/j.jcp.2009.01.006

[20] Xiu D, Karniadakis GE. The Wiener–Askey Polynomial Chaos for Stochastic Differential Equations. SIAM J Sci Comput. 2002;24(2):619–44. 10.1137/S1064827501387826

[21] Perkó Z, Gilli L, Lathouwers D, Kloosterman JL. Grid and basis adaptive polynomial chaos techniques for sensitivity and uncertainty analysis. J Comput Phys. 2014;260:54–84. 10.1016/j.jcp.2013.12.025

[22] Eldred MS, Burkardt J. Comparison of Non-Intrusive Polynomial Chaos and Stochastic Collocation Methods for Uncertainty Quantification. In: Proceedings of the 47th AIAA Aerospace Sciences Meeting. AIAA-2009-0976; 2009.

[23] Sobol′ IM. Global sensitivity indices for nonlinear mathematical models and their Monte Carlo estimates. Math Comput Simulation. 2001;55:271–280. 10.1016/S0378-4754(00)00270-6

[24] Sudret B. Global sensitivity analysis using polynomial chaos expansions. Reliab Eng Syst Safety. 2008;93(7):964–979. 10.1016/j.ress.2007.04.002

[25] Crestaux T, Le Maître O, Martinez JM. Polynomial chaos expansion for sensitivity analysis. Reliab Eng Syst Safety. 2009;94(7):1161–1172. 10.1016/j.ress.2008.10.008

[26] Gerstner T, Griebel M. Numerical integration using sparse grids. Numer Algorithms. 1998;18(3-4):209–232. 10.1023/A:1019129717644

[27] Curtis CF, Fear EC. Characterizing the point spread function of a near field ultrawideband monostatic radar imaging system. In: Radio Science Meeting (Joint with AP-S Symposium), 2013 USNC-URSI; 2013. p. 179–179.

[28] Curtis C, Fear E. Coherent summation of monostatic radar signals. In: Antennas and Propagation (EuCAP), 2013 7th European Conference on; 2013. p. 628–629.

[29] Curtis C, Fear E. Beamforming in the frequency domain with applications to microwave breast imaging. In: The 8th European Conference on Antennas and Propagation (EuCAP 2014); 2014. p. 72–76.

[30] Elahi MA, Curtis CF, Jones E, Glavin M, Fear EC, O’Halloran M. Detailed evaluation of artifact removal algorithms for radar-based microwave imaging of the breast. In: Radio Science Meeting (Joint with AP-S Symposium), 2015 USNC-URSI; 2015. p. 307–307.

[31] Kurrant D, Bourqui J, Curtis C, Fear E. Evaluation of 3D acquisition surfaces for radar-based microwave breast imaging. IEEE Trans Antennas Propag. 2015;63(11):4910–4920. 10.1109/TAP.2015.2476415

[32] Garrett J, Fear E. A New Breast Phantom With a Durable Skin Layer for Microwave Breast Imaging. IEEE Trans Antennas Propag. 2015;63(4):1693–1700. 10.1109/TAP.2015.2393854

[33] Wiggett WS, Louw M, Karusseit VOL. The histology of peau d’orange in breast cancer—what are the implications for surgery? S Afr j surg. 2012;50:75–78. 22856439 10.7196/sajs.1103

[34] Bourqui J, Okoniewski M, Fear EC. Balanced Antipodal Vivaldi Antenna With Dielectric Director for Near-Field Microwave Imaging. IEEE Trans Antennas Propag. 2010;58(7):2318–2326. 10.1109/TAP.2010.2048844

[35] Kurrant D, Fear EC. Defining Regions of Interest for Microwave Imaging Using Near-Field Reflection Data. IEEE Trans Microw Theory Techn. 2013 5;61:2137–2145. 10.1109/TMTT.2013.2250993

