# Supplementary figures and images for: Estimating the Effective Permittivity for Reconstructing Accurate Microwave-Radar Images
Source: PLoS One. 2016 Sep 9;11(9):e0160849. doi: 10.1371/journal.pone.0160849 (PMC5017770; doi:10.1371/journal.pone.0160849)

# Model #1-a

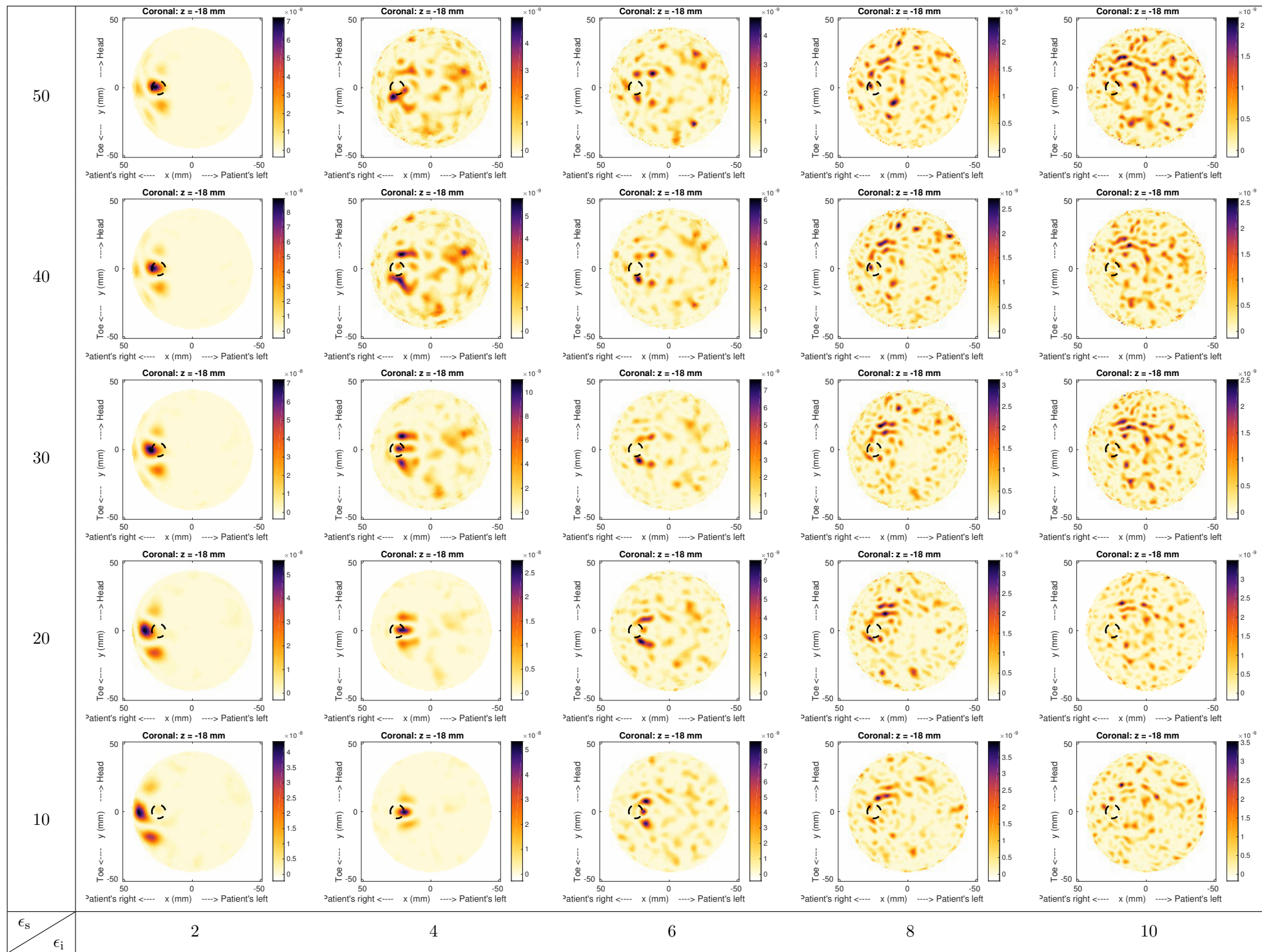

# Model #1-b

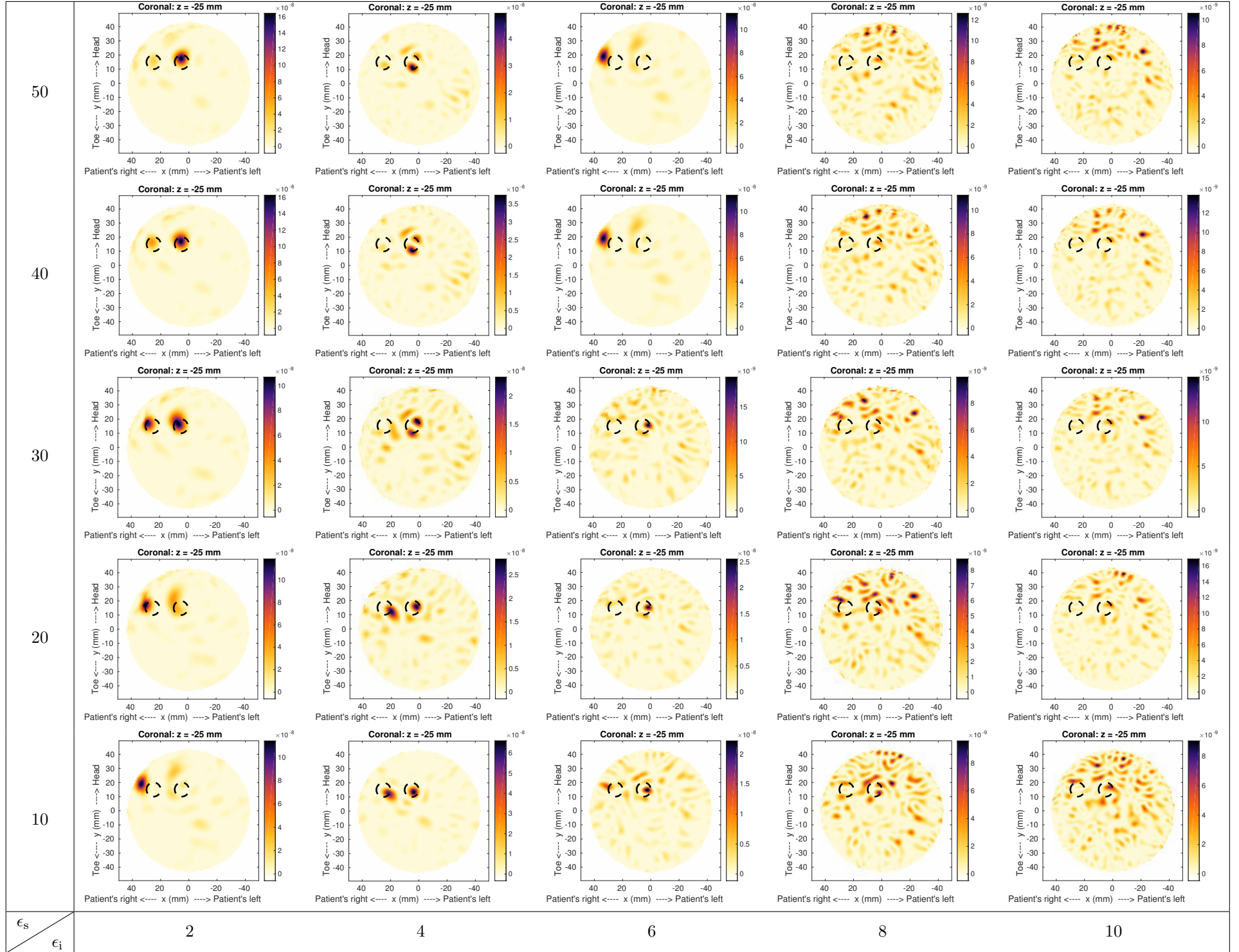

# Model #1-c

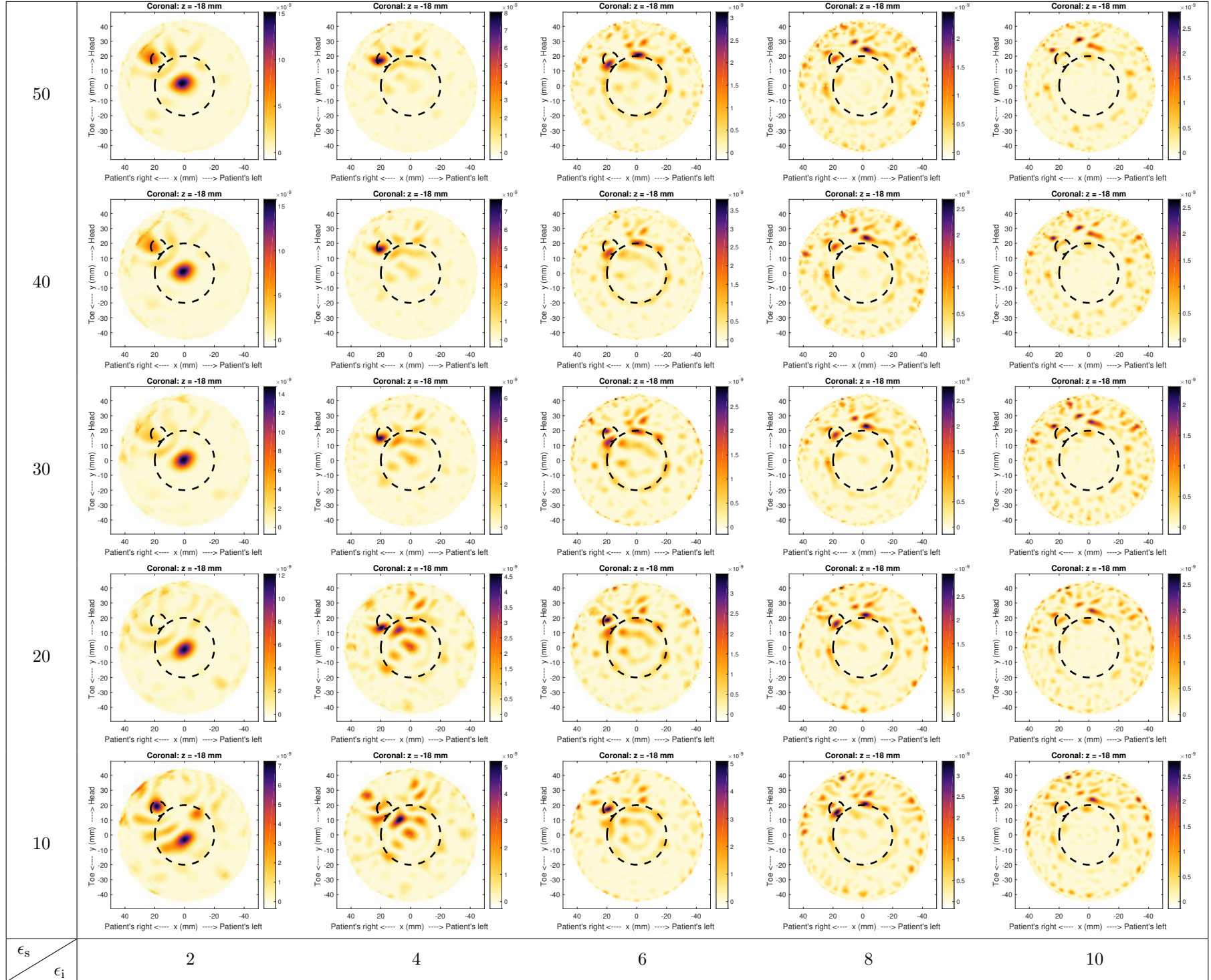

# Model #1-d

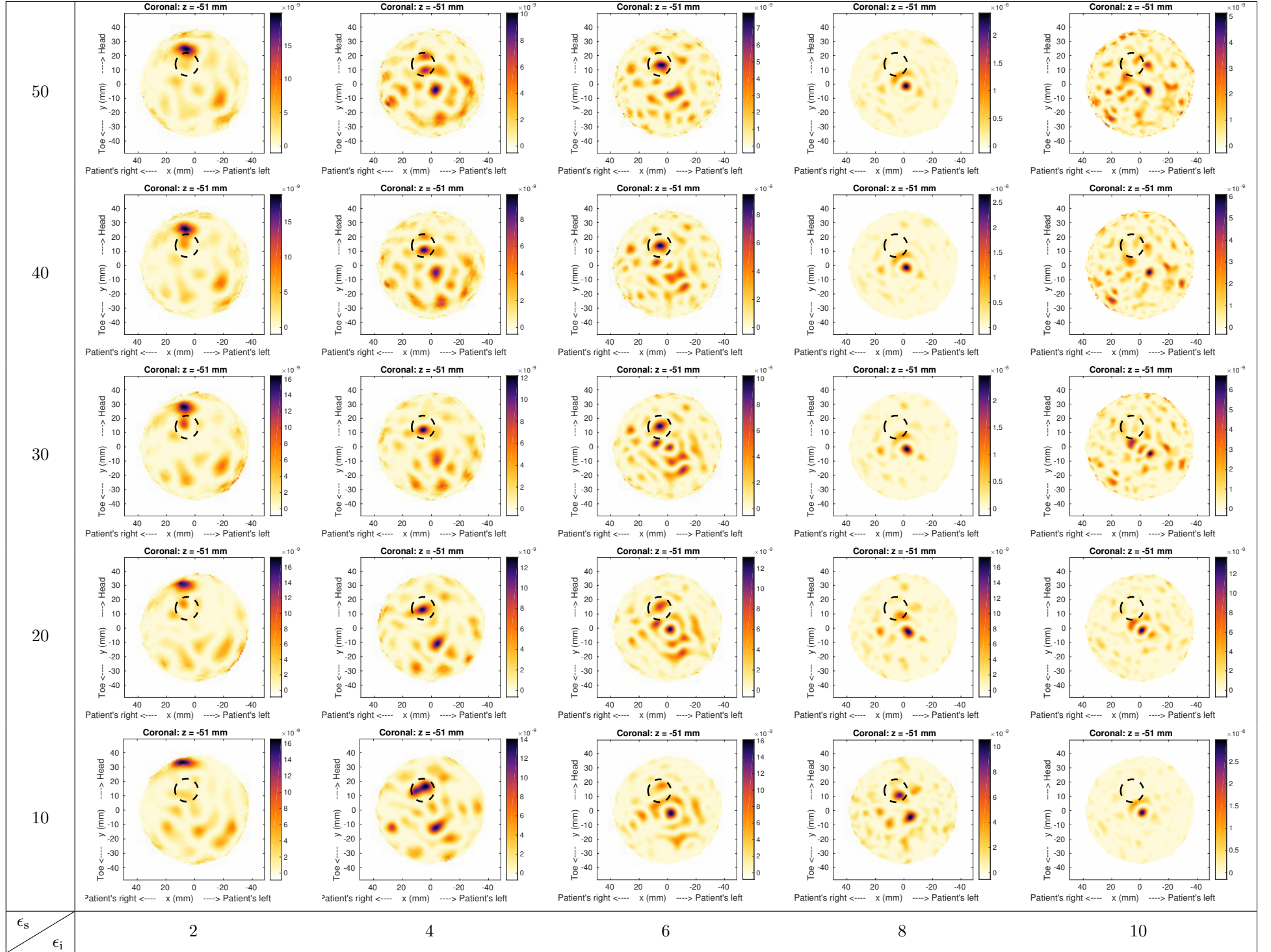

# Model #1-e

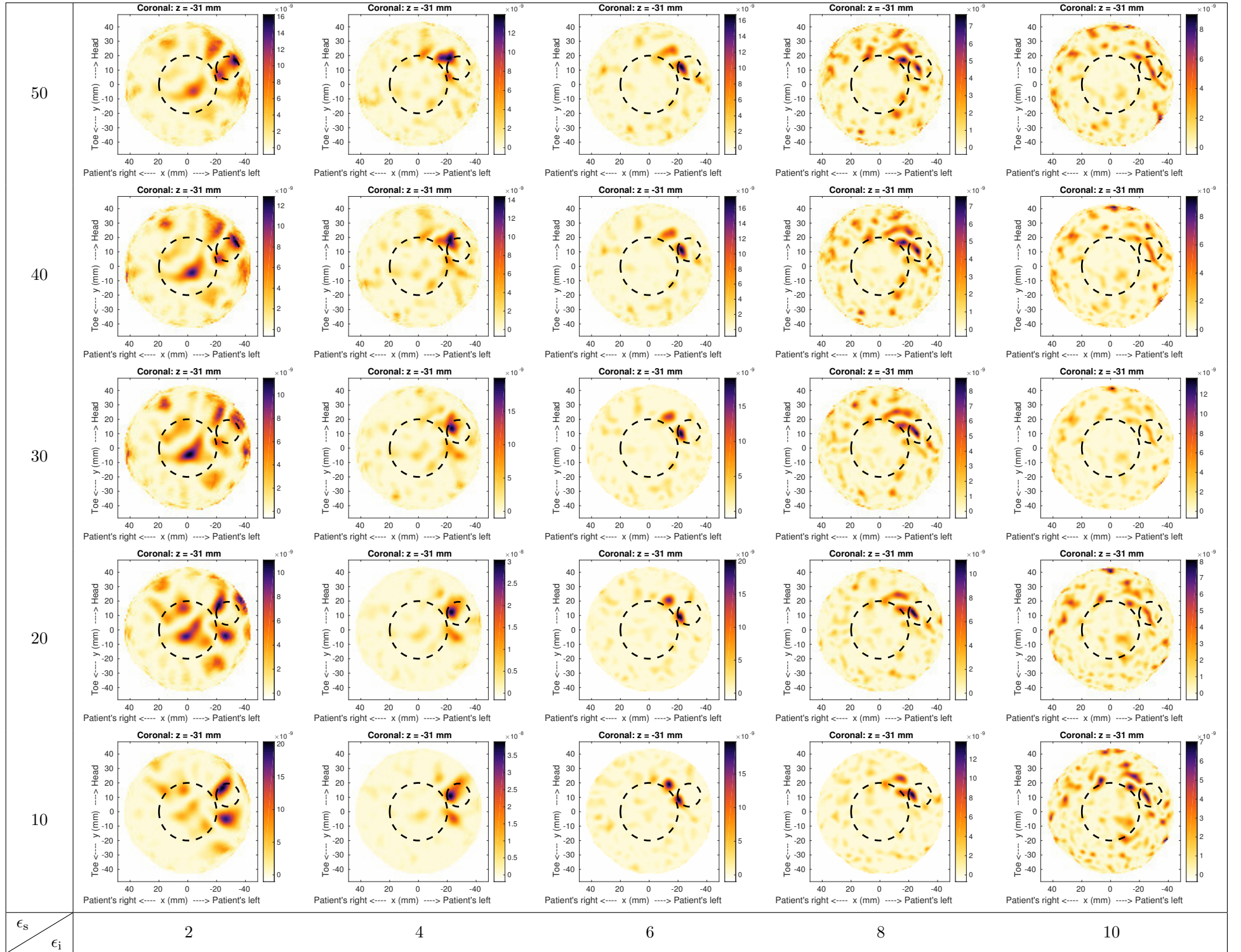

# Model #2-a

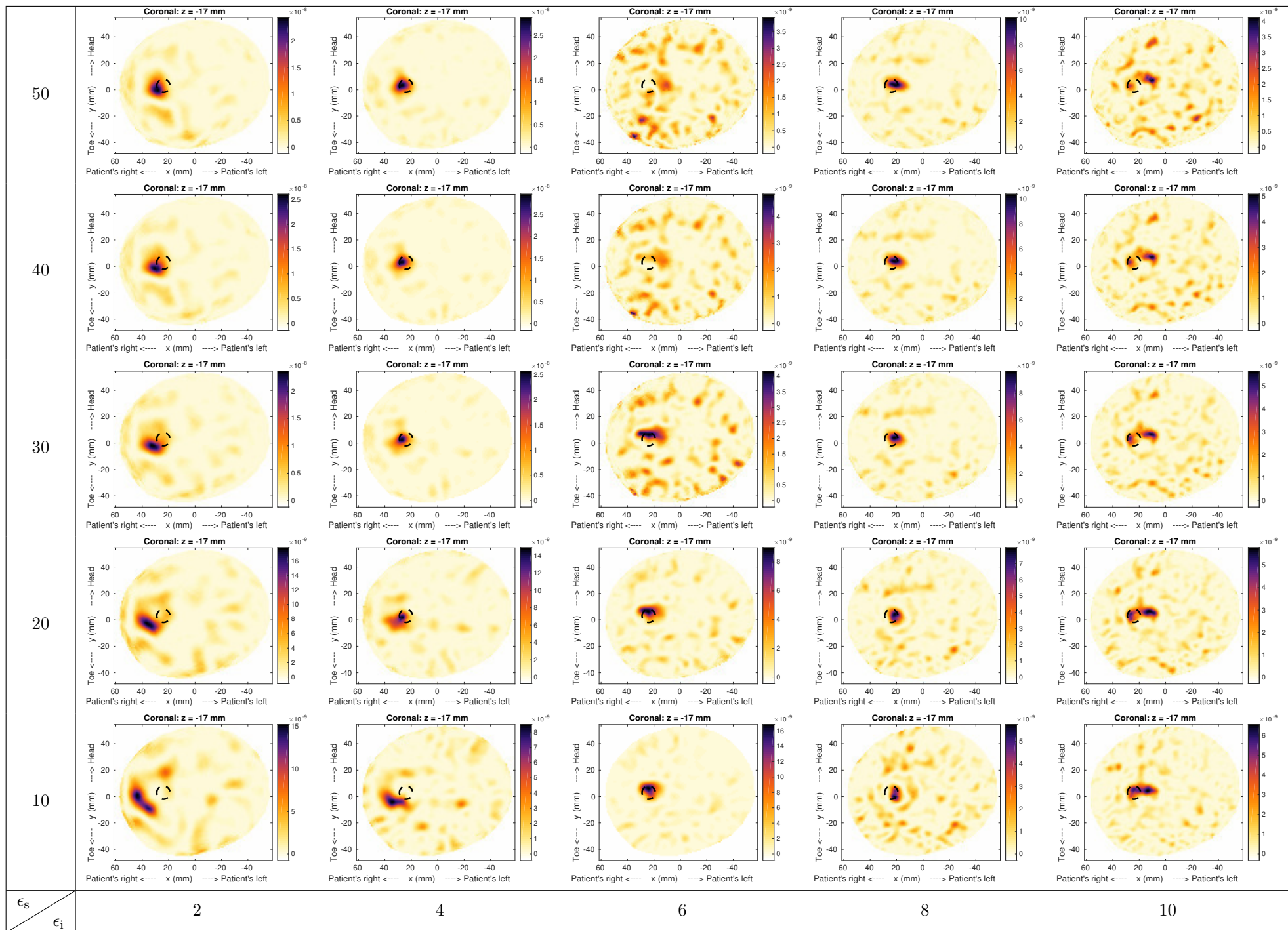

# Model #2-b

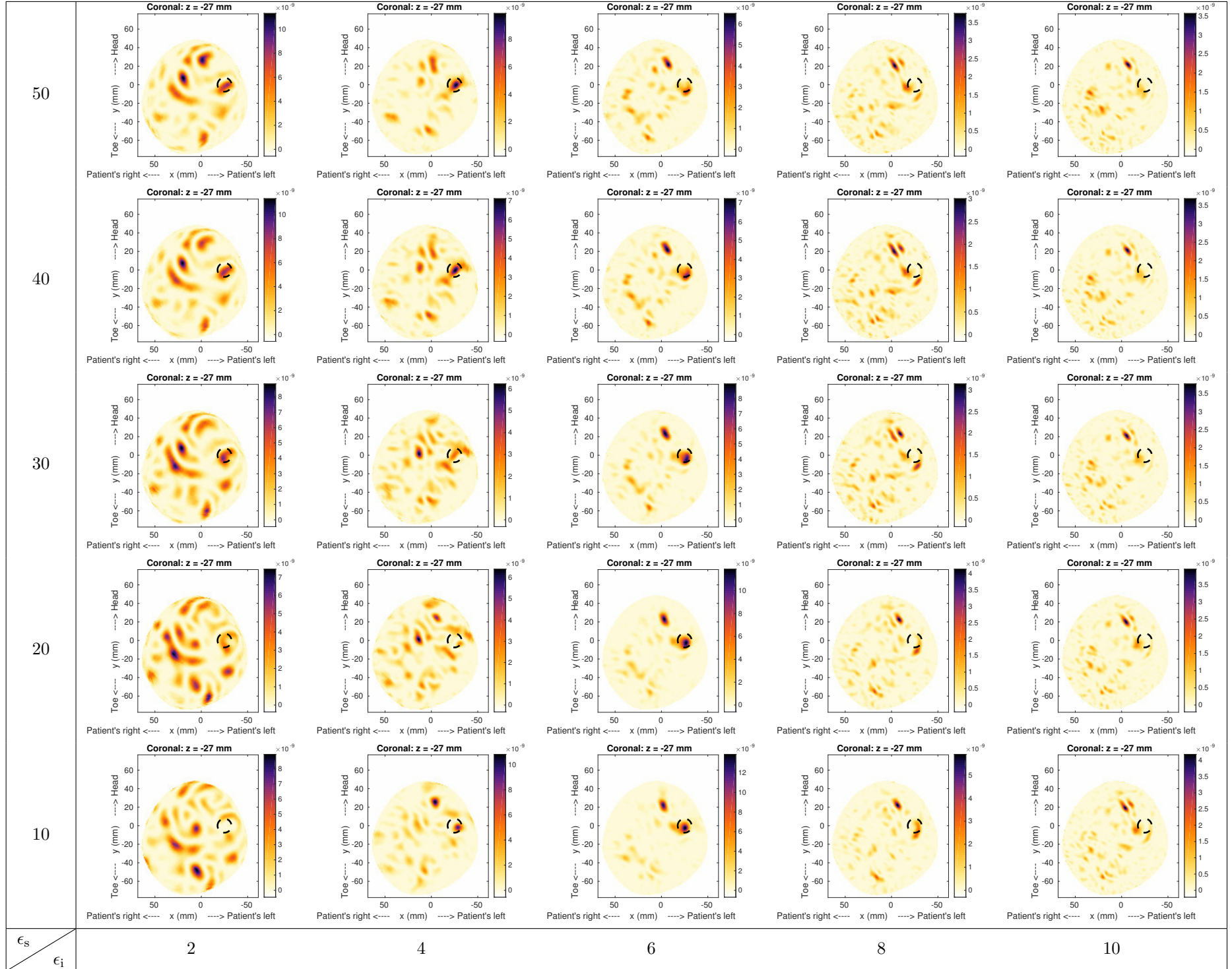

Supplement: S1 Fig — These figures show how the quality of reconstructed images depends on the permittivity estimate used. (PDF) [file pone.0160849.s001.pdf]
